# Cervical *Gardnerella vaginalis* in women with preterm prelabor rupture of membranes

**DOI:** 10.1371/journal.pone.0245937

**Published:** 2021-01-22

**Authors:** Marian Kacerovsky, Lenka Pliskova, Radka Bolehovska, Daniel Lesko, Romana Gerychova, Petr Janku, Petr Matlak, Ondrej Simetka, Jaroslav Stranik, Tomas Faist, Jan Mls, Peter Vescicik, Bo Jacobsson, Ivana Musilova

**Affiliations:** 1 Department of Obstetrics and Gynecology, University Hospital Hradec Kralove, Charles University Faculty of Medicine in Hradec Kralove, Hradec Kralove, Czech Republic; 2 Biomedical Research Center, University Hospital Hradec Kralove, Hradec Kralove, Czech Republic; 3 Institute of Clinical Biochemistry and Diagnostics, University Hospital Hradec Kralove, Hradec Kralove, Czech Republic; 4 Department of Obstetrics and Gynecology, University Hospital Brno, Faculty of Medicine Masaryk University, Brno, Czech Republic; 5 Department of Obstetrics and Gynecology, University Hospital Ostrava, Ostrava, Czech Republic; 6 Department of Obstetrics and Gynecology, Institute of Clinical Science, Sahlgrenska Academy, Gothenburg University, Gothenburg, Sweden; 7 Department of Obstetrics and Gynecology, Region Västra Götaland, Sahlgrenska University Hospital, Gothenburg, Sweden; 8 Department of Genetics and Bioinformatics, Domain of Health Data and Digitalisation, Institute of Public Health, Oslo, Norway; University of Mississippi Medical Center, UNITED STATES

## Abstract

**Objective:**

To determine the association between microbial invasion of the amniotic cavity (MIAC) and/or intra-amniotic inflammation (IAI) and the cervical prevalence of *Gardnerella vaginalis* DNA in pregnancies with preterm prelabor rupture of membrane (PPROM).

**Method:**

In total, 405 women with singleton pregnancies complicated with PPROM were included. Cervical fluid and amniotic fluid samples were collected at the time of admission. Bacterial and *G*. *vaginalis* DNA were assessed in the cervical fluid samples using quantitative PCR technique. Concentrations of interleukin-6 and MIAC were evaluated in the amniotic fluid samples. Loads of *G*. *vaginalis* DNA ≥ 1% of the total cervical bacterial DNA were used to define the cervical prevalence of *G*. *vaginalis* as abundant. Based on the MIAC and IAI, women were categorized into four groups: with intra-amniotic infection (both MIAC and IAI), with sterile IAI (IAI without MIAC), with MIAC without IAI, and without either MIAC or IAI.

**Results:**

The presence of the abundant cervical *G*. *vaginalis* was related to MIAC (with: 65% vs. without: 44%; *p* = 0.0004) but not IAI (with: 52% vs. without: 48%; *p* = 0.70). Women with MIAC without IAI had the highest load of the cervical *G*. *vaginalis* DNA (median 2.0 × 10^4^ copies DNA/mL) and the highest presence of abundant cervical *G*. *vaginalis* (73%).

**Conclusions:**

In women with PPROM, the presence of cervical *G*. *vaginalis* was associated with MIAC, mainly without the concurrent presence of IAI.

## Introduction

Rupture of the fetal membranes before the onset of regular uterine activity prior to gestational age (37 weeks) is termed preterm prelabor rupture of membranes (PPROM) [1, 2]. PPROM is not associated only with leakage of amniotic fluid, but also with the opening of a direct communication between the usually sterile intra-amniotic and non-sterile vaginal/cervical environments. Therefore, PPROM represents a serious pregnancy complication that affects ~2–4% of all pregnancies and is responsible for one-third of all preterm deliveries [1, 2].

The pathophysiology of PPROM has not been completely understood. Traditionally, the presence of microorganisms in the amniotic fluid [microbial invasion of the amniotic cavity (MIAC)] and/or elevation of inflammatory mediators in amniotic fluid [intra-amniotic inflammation (IAI)] have been considered the main cause of PPROM [1–4]. Recently, other non-infectious mechanisms such as senescence of the fetal membranes [5], and an activation of the thrombin pathway [6] due to placental abruption or subchorionic hematoma, have been suggested to be underlying causes of PPROM. The importance of these non-infectious underlying causes of PPROM is supported by the fact that MIAC and/or IAI are present (at the time of diagnosis of PPROM) only in about one third of pregnancies with PPROM [7–9]. However, a comprehensive understanding of the pathophysiology of PPROM and its intra-amniotic complications appears to be a crucial step for the further development of effective preventive measures and optimal management strategies.

Cervicovaginal microbiota is a unique microbial niche of the human body due to the dominant presence of *Lactobacillus* species, such as *L*. *crispatus*, *L*. *gasseri*, *L*. *iners*, and *L*. *jensenii*, in most of the women [10, 11]. In addition, cervicovaginal microbiota play an important role in the maintenance of the epithelial barrier integrity of the cervix [12, 13], and the regulation of the ascension of microorganisms from the vagina/cervix to the uterine cavity and subsequently into the amniotic cavity [14, 15]. In women with PPROM, the dominant presence of the cervical *L*. *crispatus* has been shown to be associated with lower frequencies of MIAC and intra-amniotic infection (the presence of both MIAC and IAI) [14]. Conversely, non*-Lactobacillus* dominated microbiota in the cervix, characterized by high bacterial diversity, was related to a higher prevalence of IAI [15].

*Gardnerella vaginalis*, a facultative anaerobic bacterium, represents the most typical microbe associated with bacterial vaginosis [16]. Nevertheless, *G*. *vaginalis* can be found in both *Lactobacillus*-dominated and non-*Lactobacillus*-dominated cervicovaginal microbiota [17, 18]. Despite the well-established relationship between bacterial vaginosis, with reported presence of *G*. *vaginalis* as one of the microbial agents, and PPROM [19–21], evidence is lacking to ascertain whether the cervical presence *G*. *vaginalis* could affect the development of intra-amniotic complications, such MIAC and/or IAI, in women with PPROM.

Therefore, this study was conducted to determine an association between the presence of the cervical *G*. *vaginalis* DNA and MIAC and/or IAI in PPROM pregnancies. We also assessed the association between the presence of cervical *G*. *vaginalis* DNA and short-term neonatal morbidity.

## Material and methods

This prospective cohort study of pregnant women between 24^+0^ and 36^+6^ weeks’ gestation, who were admitted to the Department of Obstetrics and Gynecology of the University Hospital Hradec Kralove in the Czech Republic, was conducted between August 2015 and January 2020. The inclusion criteria were: i) singleton pregnancies complicated by the presence of PPROM and ii) age ≥ 18 years. The exclusion criteria were as follows: i) fetal growth restriction; ii) congenital or chromosomal fetal abnormalities; iii) gestational or pregestational diabetes; iv) gestational hypertension; v) preeclampsia; vi) signs of fetal hypoxia; and vii) significant vaginal bleeding.

Gestational age was established using first-trimester fetal biometry. PPROM was diagnosed by the confirmation of the pooling of amniotic fluid in the vagina during the examination with a sterile speculum. If clinical doubts about PPROM remained after this examination, amniotic fluid leakage was confirmed by the presence of insulin-like growth factor binding proteins (Actim PROM test; Medix Biochemica, Kauniainen, Finland) in the vaginal fluid. Treatment of PPROM was described in our previous studies[14, 22].

In total, 311 women from this cohort were used in our previous study based on the presence of *L*. *crispatus-* and *L*. *iners-*dominated cervical microbiota in women with PPROM [14].

Women’s selection and sampling procedures were performed in accordance with the Declaration of Helsinki and applicable local regulatory requirements after approval from the Ethics Committee of the University Hospital Hradec Kralove (July 2014; No 201408 S07P). Written informed consents were obtained from all participants. All participants in the study were Caucasian.

### Cervical sampling

Cervical fluid samples were collected prior to administration of corticosteroids, antibiotics, or tocolytics using a Dacron polyester swab placed in the cervical canal for 20 s to achieve saturation. Upon collection, the Dacron polyester swab was inserted into a polypropylene tube containing 1.5 mL of phosphate-buffered saline. Each tube was shaken for 20 min, followed by centrifugation at 300 × *g* for 15 min at room temperature. The supernatant and pellet were aliquoted and stored at -80°C until further analyses. The pellet was used to assess the presence of bacterial and *G*. *vaginalis* DNA.

### Amniotic fluid sampling

Ultrasonography-guided transabdominal amniocentesis was performed prior to administering corticosteroids, antibiotics, or tocolytics. Details about amniotic fluid sampling have been described in our previous publication [14].

### Detection of cervical G. vaginalis DNA

Nucleic acid was isolated from the pellets using the tissue protocol with the QIAamp DNA Mini Kit (Qiagen, Hilden, Germany) [14]. In-house real-time PCR was developed to detect *G*. *vaginalis*. Primers and hydrolysis probes for *G*. *vaginalis* were designed from 16S rRNA region to amplify a 164 bp amplicon (forward primer GV F578 GTG AAA GCC CAT CGC TTA AC, reverse primer GV R741 TTC GCT TCT CAG CGT CAG TA and probe FAM-BHQ1 AAT TCT CGG TGT AAC GGT GG) and real-time PCR was performed on a Rotor-Gene Q instrument (Qiagen) in 25 μL reaction volumes, containing universal 2x gb IPC PCR Master Mix (Generi Biotech, Hradec Kralove, Czech Republic) with an internal positive control, primers at concentrations of 400 nM each, and dual-labeled hydrolysis probes (FAM-BHQ1) at a concentration of 200 nM [14]. Primers and probes were synthesized by Generi Biotech. Amplification parameters were as follows: 95°C for 5 min, followed by 45 cycles of 95°C for 15 s and 60°C for 30 s [14]. PCR detection was performed by absolute quantification and a standard curve was generated from serial 10-fold dilutions of linearized and normalized plasmids containing the cloned target sequences for *G*. *vaginalis* at concentrations of 10^7^ copies/μL (Generi Biotech, Hradec Kralove, Czech Republic).

To identify the relative abundance of *G*. *vaginalis*, total bacterial DNA detection was performed using quantitative RT-PCR–BactQuant [23]. To quantify the bacterial load, we used the forward primer CCT ACG GGD GGC WGC A, reverse primer GGA CTA CHV GGG TMT CTA ATC, and hydrolysis probe FAM-BHQ1 CAG CCG CGG TA, and a calibration curve was generated using 10-fold dilutions of linearized and normalized plasmid containing the cloned target sequence of the 466-bp region in the V3-V4 domain of 16S rRNA at a concentration of 10^7^ copies/μL (Generi Biotech) [14]. RT-PCR for *G*.*vaginalis* detection was validated with a commercially available positive control Amplirun Gardnerella vaginalis DNA Control (Vircell Microbiologists, Spain).

Relative abundance of *G*. *vaginalis* in cervical microbiota was expressed as the ratio of the DNA quantity of *G*. *vaginalis* to the total cervical bacterial DNA load. The PCR conditions used in the BactQuant assay were the same as those used for *G*. *vaginalis*.

### Detection of Ureaplasma spp., Mycoplasma hominis, and Chlamydia trachomatis and others bacteria in amniotic fluid

DNA was isolated from the amniotic fluid using a QIAamp DNA mini kit according to the manufacturer’s instructions.

RT-PCR was performed on a Rotor-Gene 6000 instrument using the commercial AmpliSens^®^ *C*. *trachomatis*/*Ureaplasma*/*M*. *hominis*-FRT kit (Federal State Institution of Science, Central Research Institute of Epidemiology, Moscow, Russia) to detect the DNA from *Ureaplasma* spp., *M*. *hominis*, and *C*. *trachomatis* in the same PCR tube. As a control, we amplified beta-actin, a housekeeping gene, to exclude the presence of PCR inhibitors.

Bacterial DNA was identified by PCR targeting 16S rRNA using the following primers: 5ʹ-CCAGACTCCTACGGGAGGCAG-3ʹ (V3 region) and 5ʹ-ACATTTCACAACACGAGCTGACGA-3ʹ (V6 region) [24]. The products were visualized on an agarose gel. Positive reactions yielded 950 bp products that were subsequently analyzed using sequencing. The 16S PCR products were purified and sequenced using PCR with the above primers and BigDye Terminator kit v3.1 (Thermo Fisher Scientific, Waltham, MA). The bacteria were then typed using the sequences obtained from BLAST^®^ and SepsiTest^TM^ BLAST.

### Aerobic and anaerobic cultures of the amniotic fluid

The amniotic fluid samples were cultured in Columbia agar with sheep’s blood, *G*. *vaginalis* selective medium, MacConkey agar, *Neisseria*-selective medium (modified Thayer–Martin medium), Sabouraud agar, and Schaedler anaerobe agar. The plates were cultured for 6 days and checked daily. The species were identified by matrix-assisted laser desorption/ionization time-of-flight mass spectrometry using MALDI Biotyper software (Bruker Daltonics, Billerica, MA).

### Concentration of interleukin-6 in amniotic fluid

Concentrations of interleukin (IL)-6 in the amniotic fluid samples obtained between August 2015 to December 2018 were assessed using a Milenia QuickLine IL-6 lateral flow immunoassay using a Milenia POCScan reader (Milenia Biotec, GmbH, Giessen, Germany). The measurement range was 50–10,000 pg/mL. The intra- and inter-assay variations were 12.1% and 15.5%, respectively [25].

Concentration of IL-6 in the amniotic fluid samples taken between December 2018 and January 2020 were evaluated using automated electrochemiluminescence immunoassay method with immuno-analyzer Cobas e602, which is a part of the Cobas 8000 platform (Roche Diagnostics, Basel, Switzerland). The measurement range was 1.5–5,000 pg/mL, which could be extended to 50,000 pg/mL with a 10-fold dilution of the sample. The coefficients of variation for inter- and intra-assay precisions were < 10% [22].

### Definition of G. vaginalis as abundant bacteria in cervical microbiota

*G*. *vaginalis* was classified as abundant bacteria in cervical microbiota when its relative abundance was > 1% of the total cervical microbiota [26–29] (cervical loads of *G*. *vaginalis* DNA > 1% of the amount of the cervical bacterial DNA).

### Diagnosis of MIAC

MIAC was defined as the presence of microorganisms detected by culture and/or the detection of microbial nucleic acids in amniotic fluid (PCR analysis of *Ureaplasma* spp., *M*. *hominis*, *C trachomatis* and PCR analysis of 16S rRNA gene). Due to heterogeneity of microorganisms present in amniotic fluid obtained from PPROM pregnancies, with Ureaplasma spp. being the most common, [30, 31] women with MIAC were further split into three groups: i) with just *Ureaplasma* spp. in the amniotic fluid; ii) with the bacteria other than *Ureaplasma* spp. in the amniotic fluid; and iii) with both *Ureaplasma* spp. and the other bacteria in the amniotic fluid.

### Diagnosis of IAI

IAI in PPROM pregnancies was defined as amniotic fluid IL-6 concentrations ≥ 745 pg/mL, when IL-6 was measured using lateral flow-based immunoassay point-of-care test [32, 33], or IL-6 ≥ 3000 pg/mL, when IL-6 was measured using automated electrochemiluminescence immunoassay method [22].

Based on the presence of MIAC and/or IAI, the women were categorized into four groups: with intra-amniotic infection (both MIAC and IAI), with sterile IAI (IAI without MIAC), with MIAC without IAI, and without either MIAC or IAI.

### Definitions of select aspects of short-term neonatal morbidity

Maternal and perinatal medical records were reviewed by seven investigators (DL, MK, JS, TF, JM, PV, and IM). Data regarding short-term neonatal morbidity were reviewed for all newborns. “Compound neonatal morbidity” was defined in this study as follows: the need for intubation, and/or need for nasal continuous positive airway pressure, and/or respiratory distress syndrome (defined by the presence of two or more of the following criteria: evidence of respiratory compromise, persistent oxygen requirement for more than 24 h, administration of exogenous surfactant, radiographic evidence of hyaline membrane disease); and/or transient tachypnea of newborns (defined as any oxygen supplement requirement during the first 6 h that did not increase during the subsequent 18 h as clinical conditions improved within 3–6 h and chest radiographs was either normal or indicating reduced translucency, infiltrates, and hyperinsufflation of the lungs); and/or bronchopulmonary dysplasia (defined as infant oxygen requirement at 28 days of age); and/or pneumonia (diagnosed by abnormal findings on chest X rays); and/or retinopathy of prematurity (identified using retinoscopy); and/or intraventricular hemorrhage (diagnosed by cranial ultrasound examinations according as described by Papile et al. [34]); and/or necrotizing enterocolitis (defined as radiologic findings of either intramural gas or free intra-abdominal gas); and/or early- (during the first 72 h of life) or late-onset- (between the ages of 4 and 120 days) sepsis (either proven by bacterial culture or clinically highly suspected sepsis); and/or neonatal death before hospital discharge.

### Statistical analyses

The normality of the data was tested using the D’Agostino–Pearson omnibus normality test and Shapiro–Wilk test. Continuous variables were compared using nonparametric Mann-Whitney *U* test or Kruskal-Wallis test, and presented as the median value (interquartile range [IQR]). Categorical variables were compared using Fisher’s exact test or chi-square test, and presented as a number (%). A partial correlation was used to adjust the results for the different methods of IL-6 assessment. Differences were considered significant at *p* < 0.05. All *p* values were obtained from two-tailed tests, and all statistical analyses were performed using GraphPad Prism version 6.0h software for Mac OS X (GraphPad, Inc., San Diego, CA, USA) and SPSS version 19.0 for Mac OS X (SPSS, Inc., Chicago, IL, USA).

## Results

A total of 464 women with singleton pregnancies at a gestational age of 24+0 to 36+6 weeks were admitted during the study period. A total of 24 women were excluded because their cervical fluid was not collected (n = 15), amniocentesis failure (n = 3), unconfirmed PPROM (n = 2), and delivery prior to amniocentesis (n = 4). In total, 35 women were subsequently excluded due the following medical reasons: gestational diabetes mellitus (n = 11), fetal growth restriction (n = 8), chronic hypertension (n = 5), pre-gestation diabetes mellitus (n = 4), preeclampsia (n = 4), and pregnancy induced hypertension (n = 3). A total of 405 women were included in the study.

MIAC and IAI were identified in 24% (96/405) and 18% (73/405) of the women, respectively. *G*. *vaginalis* DNA in amniotic fluid was detected in 1% (4/405) of the women. The women’s demographic and clinical data based on the presence and absence of MIAC and IAI are shown in Table 1. Intra-amniotic infection was observed in 12% (47/405), sterile intra-amniotic inflammation in 6% (26/405), MIAC without IAI in 12% (49/405), and the absence of either MIAC or IAI in 70% (283/405) of the women. Bacterial species identified in the amniotic fluid obtained in women with intra-amniotic infection and with MIAC without IAI are shown in Table 2.

**Table 1:** Maternal and clinical characteristics of pregnancies complicated by preterm prelabor rupture of the membranes.

Characteristic	Women with PPROM (n = 405)
Maternal age [years, median (IQR)]	31 (27–34)
Primiparous [number (%)]	232 (57%)
Pre-pregnancy body mass index [kg/m^2^, median (IQR)]	23.8 (21.0–27.3)
Smoking [number (%)]	48 (12%)
Interval between PPROM and amniocentesis [hours, median (IQR)]	4 (3–8)
Gestational age at admission [weeks, median (IQR)]	34+0 (31+3–35+3)
Gestational age at delivery [weeks, median (IQR)]	34+2 (32+3–35+5)
Latency between PPROM and delivery [hours, median (IQR)]	46 (15–120)
CRP levels at admission [mg/L, median (IQR)]	5.1 (2.7–9.4)
WBC count at admission [x10^9^ L, median (IQR)]	12.3 (10.1–14.9)
Administration of antibiotics [number (%)]	361 (89%)
Administration of corticosteroids [number (%)]	263 (65%)
Spontaneous vaginal delivery [number (%)]	275 (68%)
Cesarean delivery [number (%)]	124 (31%)
Forceps delivery [number (%)]	6 (1%)
Birth weight [grams, median (IQR)]	2240 (1760–2605)
Apgar score <7; 5 minutes [number (%)]	15 (4%)
Apgar score <7; 10 minutes [number (%)]	5 (1%)

Abbreviations

CRP: C-reactive protein

IAI: intra-amniotic inflammation

IQR: interquartile range

MIAC: microbial invasion of the amniotic cavity

PPROM: preterm prelabor rupture of membranes

WBC: white blood cells

Continuous variables are presented as median (IQR) and categorical as number (%).

**Table 2:** Bacterial species identified in the amniotic fluid obtained during the initial amniocentesis from patients with preterm prelabor rupture of the membranes.

Women with intra-amniotic infection (n = 47)	Women with MIAC without IAI (n = 49)
*Ureaplasma* spp. (n = 22)	*Ureaplasma* spp. (n = 27)
*Ureaplasma* spp. + *Fusobacterium nucleatum* (n = 2)	*Ureaplasma* spp. + *Chlamydia trachomatis* (n = 1)
*Ureaplasma* spp. + *Escherichia coli* (n = 1)	*Ureaplasma* spp. + *Gardnerella vaginalis* (n = 1)
*Ureaplasma* spp. + *Enterococcus faecium* (n = 1)	*Ureaplasma* spp. + *Streptococcus mitis* (n = 1)
*Ureaplasma* spp. + *Streptococcus anginosus* (n = 1)	*Ureaplasma* spp. + *Lactobacillus jensenii* (n = 1)
*Ureaplasma* spp. + *Chlamydia trachomatis* (n = 1)	*Chlamydia trachomatis* (n = 2)
*Ureaplasma* spp. + *Dialister micraerophilus + Atopobium vaginae* (n = 1)	*Gardnerella vaginalis* (n = 1)
*Haemophilus influenzae* (n = 5)	*Haemophilus influenzae* (n = 1)
*Streptococcus ovalis* + *Streptococcus anginosus* + *Campylobacter ureolyticus* (n = 1)	*Lactobacillus crispatus* + *Enterococcus faecalis + Streptococcus salivarius* (n = 1)
*Anaerococcus tetradius* (n = 1)	*Lactobacillus iners* (n = 1)
*Fusobacterium nucleatum* (n = 1)	*Lactobacillus gasseri* (n = 1)
*Gardnerella vaginalis* (n = 1)	*Lactobacillus gasseri + Bifidobacterium breve* (n = 1)
*Lactobacillus jensenii* (n = 1)	*Lactobacillus gasseri + Gardnerella vaginalis + Corynebacterium* spp. (n = 1)
*Parvominas micra* (n = 1)	*Peptostreptococcus stomatis* (n = 1)
*Peptoniphilus* spp. (n = 1)	*Prevotella bivia* (n = 1)
*Propionibacterium acnes* (n = 1)	*Propionibacterium acnes* (n = 1)
*Streptococcus agalactiae* (n = 1)	*Sneathia sanguinegens* (n = 1)
*Streptococcus anginosus* (n = 1)	*Staphylococcus epidermidis + Dermabacter hominis* (n = 1)
*Streptococcus intermedius* (n = 1)	*Staphylococcus warneri* (n = 1)
*Sneathia sanguinegens* (n = 1)	*Streptococcus agalactiae* (n = 1)
Non-identifiable bacteria (n = 1)	*Streptococcus intermedius* (n = 1)
	*Streptococcus mitis* (n = 1)

**Fig 1:**
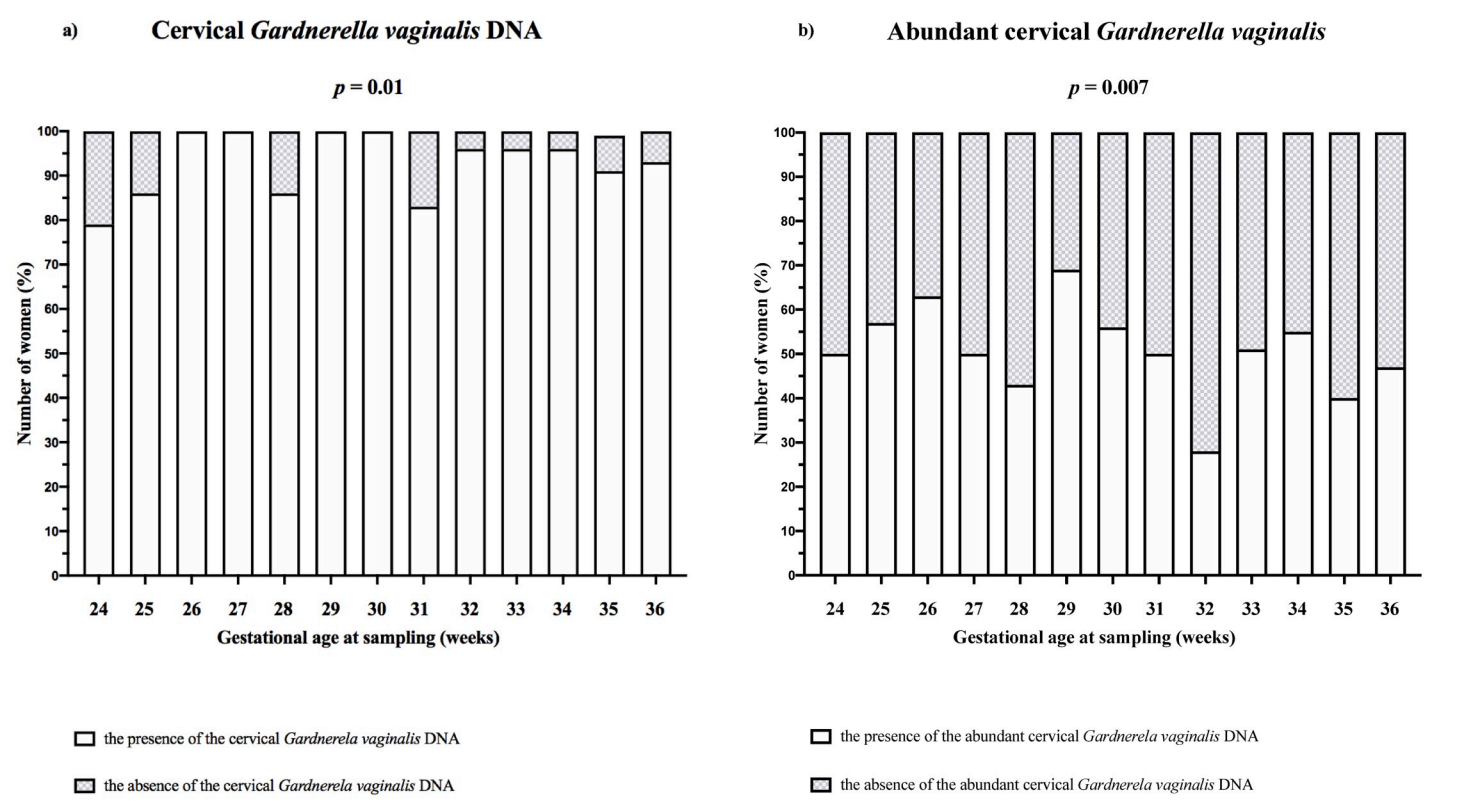
The rates of the presence of *G*. *vaginalis* DNA (a) and the presence of abundant cervical *G*. *vaginalis* (cervical microbial loads of *G*. *vaginalis* DNA ≥ 1% of amount of the cervical bacterial DNA; (b) according to gestational age at PPROM. Differences among gestational ages at sampling (weeks) and the rates of the presence of *G*. *vaginalis* DNA (*p* = 0.01) and abundant cervical *G*. *vaginalis* DNA (*p* = 0.007) were observed.

Cervical *G*. *vaginalis* DNA was found in 94% (379/405) of the women. Abundant cervical *G*. *vaginalis* (cervical loads of *G*. *vaginalis* DNA > 1% of the amount of the cervical bacterial DNA) was found in 49% (197/405) of the women. Differences between gestational age at sampling and the rates of the presence of cervical *G*. *vaginalis* DNA (*p* = 0.01, Fig 1A) and abundant cervical *G*. *vaginalis* DNA (*p* = 0.007, Fig 1B) were observed.

### MIAC and cervical G. vaginalis

The presence of cervical *G*. *vaginalis* DNA was identified in 97% (93/96) and 93% (286/309) of women with and without MIAC, respectively.

**Fig 2:**
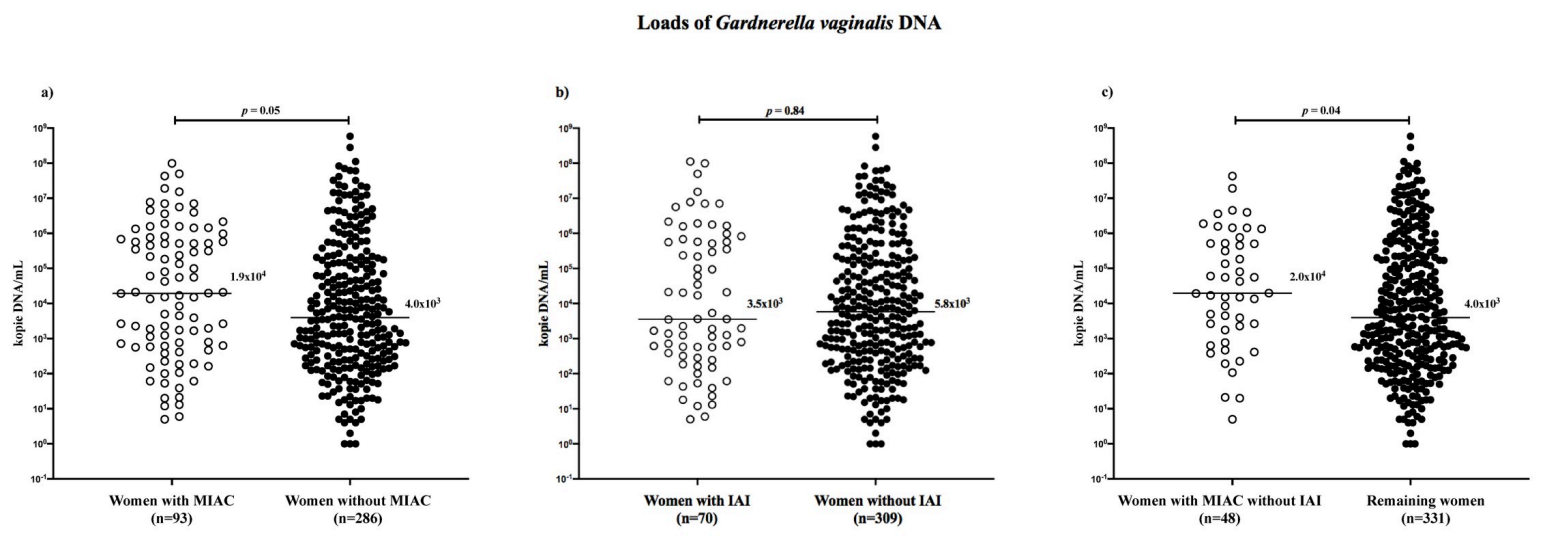
Microbial loads of *G*. *vaginalis* DNA in women with preterm prelabor rupture of the membranes with the cervical presence of *G*. *vaginalis* DNA with respect to microbial invasion of the amniotic cavity (a), intra-amniotic inflammation (b), and microbial invasion of the amniotic cavity without intra-amniotic inflammation (c).

Among women with cervical *G*. *vaginalis* DNA, no difference in the microbial loads cervical *G*. *vaginalis* DNA was found between women with and without MIAC (with MIAC: median 1.9 × 10^4^ copies DNA/mL, IQR 6.0 × 10^2^–6.3 × 10^5^ vs. without MIAC: median 4.0 × 10^3^ copies DNA/mL, IQR 2.8 × 10^2^–1.5 × 10^5^; *p* = 0.052; Fig 2A).

The presence of the abundant cervical *G*. *vaginalis* was higher in women with MIAC than in those without this complication [with MIAC: 65% (62/96) vs. without MIAC: 44% (135/309); *p* = 0.0004).

Among women with the presence of cervical *G*. *vaginalis* DNA, those with MIAC were also split into three subgroups: i) with just *Ureaplasma* spp. in the amniotic fluid (n = 49); ii) with the bacteria other than *Ureaplasma* spp. in the amniotic fluid (n = 36); and iii) with both *Ureaplasma* spp. and the other bacteria in the amniotic fluid (n = 11). No difference in the loads of cervical *G*. *vaginalis* DNA (*Ureaplasma* spp.: median 6.5 × 10^3^ copies DNA/mL, IQR 4.3× 10^2^–4.9 × 10^5^ vs. other: median 5.6 × 10^4^ copies DNA/mL, IQR 6.1 × 10^2^–1.4 × 10^6^ vs. both: median 5.6 × 10^4^ copies DNA/mL, IQR 6.3 × 10^2^–5.8 × 10^5^; *p =* 0.60) was identified among the subgroups. No difference in the rate of the presence of the abundant cervical *G*. *vaginalis* was revealed among the subgroups [with *Ureaplasma* spp. alone: 57% (28/49), the other bacteria: 72% (26/36), and both *Ureaplasma* spp. and other bacteria: 73% (8/11); *p* = 0.30].

### IAI and cervical G. vaginalis

The cervical presence of *G*. *vaginalis* DNA was identified in 96% (70/73) and 93% (309/332) of the women with and without IAI, respectively.

No difference in the loads of cervical *G*. *vaginalis* DNA was observed between the women with the presence of cervical *G*. *vaginalis* DNA with and without IAI (with IAI: median: 3.5 × 10^3^ copies DNA/mL, IQR 3.1 × 10^2^–5.7 × 10^5^ vs. without IAI: median 5.8 × 10^3^ copies DNA/mL, IQR 3.6 × 10^2^–1.9 × 10^5^; *p* = 0.84; Fig 2B).

No difference in the rates of the presence of the abundant cervical *G*. *vaginalis* was identified between the women with and without IAI [with IAI: 52% (38/73) vs. without IAI: 48% (158/332); *p* = 0.70].

### Association of cervical G. vaginalis with MIAC and/or IAI

The presence of cervical *G*. *vaginalis* DNA was identified in 96% (45/47) of the women with intra-amniotic infection, in 96% (25/26) of the women with sterile IAI, in 98% (48/49) of the women with MIAC without IAI, and in 92% (261/284) of the women without either MIAC or IAI.

**Fig 3:**
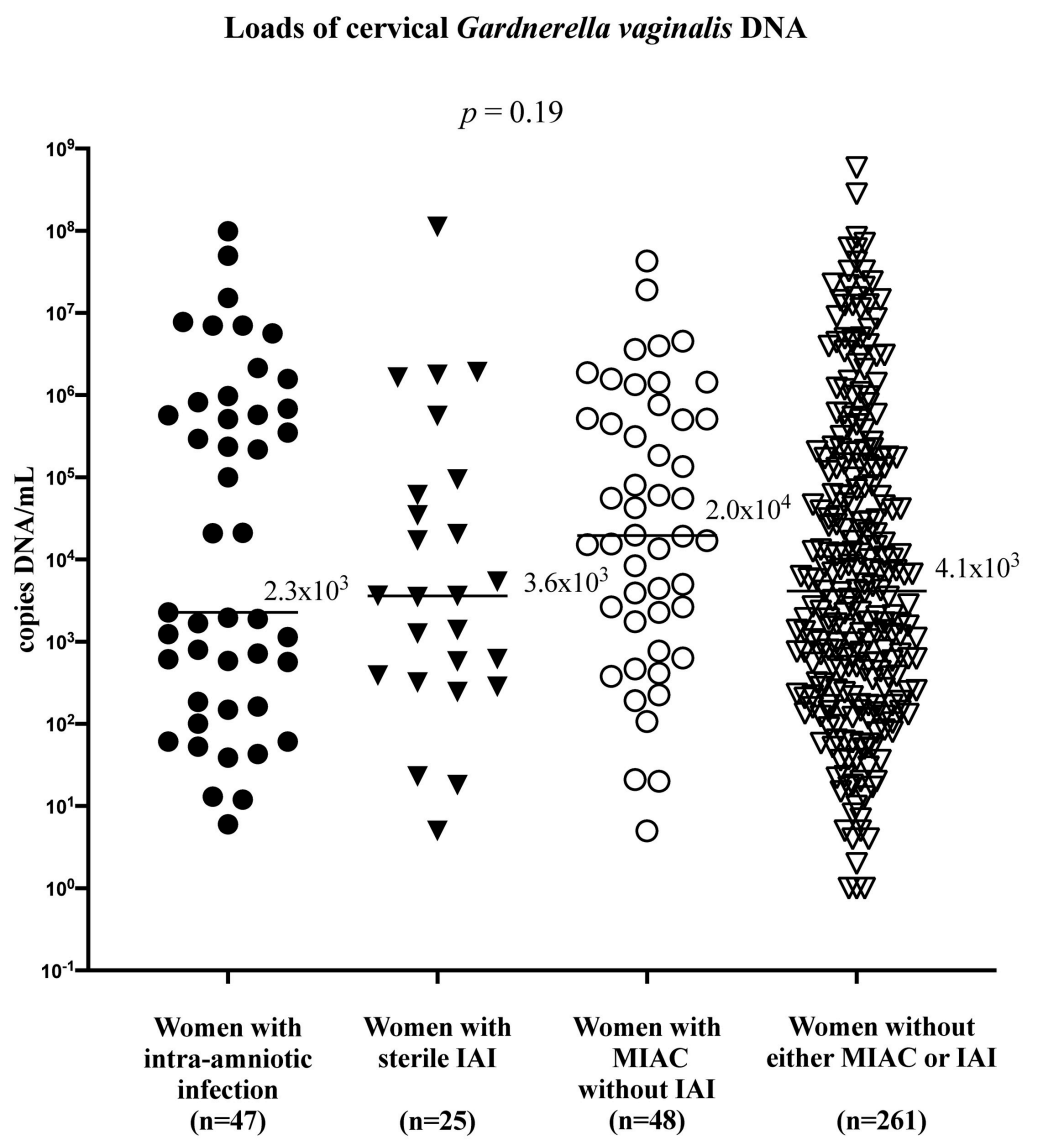
Loads of *G*. *vaginalis* DNA in women with preterm prelabor rupture of the membranes with the cervical presence of *G*. *vaginalis* DNA with respect to the presence of microbial invasion of the amniotic cavity and/or intra-amniotic inflammation. No differences in the cervical loads of G. vaginalis DNA was revealed among women with intra-amniotic infection, with sterile IAI, with MIAC without IAI and without either MIAC or IAI (*p* = 0.19). Abbreviations: IAI, intra-amniotic inflammation; MIAC, microbial invasion of the amniotic cavity.

No difference in the cervical loads of *G*. *vaginalis* DNA was revealed among women with intra-amniotic infection, with sterile IAI, with MIAC without IAI, and without either MIAC or IAI (intra-amniotic infection: median 2.3 × 10^3^ copies DNA/mL, IQR 1.7 × 10^2^–7.5 × 10^5^; sterile IAI: median 3.6 × 10^3^ copies DNA/mL, IQR 3.5 × 10^2^–7.7 × 10^4^; MIAC without IAI: median 2.0 × 10^4^ copies DNA/mL, IQR 1.9 × 10^3^–5.2 × 10^5^; without either MIAC or IAI: median 4.1 × 10^3^ copies DNA/mL, IQR 2.6 × 10^2^–1.6 × 10^5^; *p* = 0.19; Fig 3).

**Fig 4:**
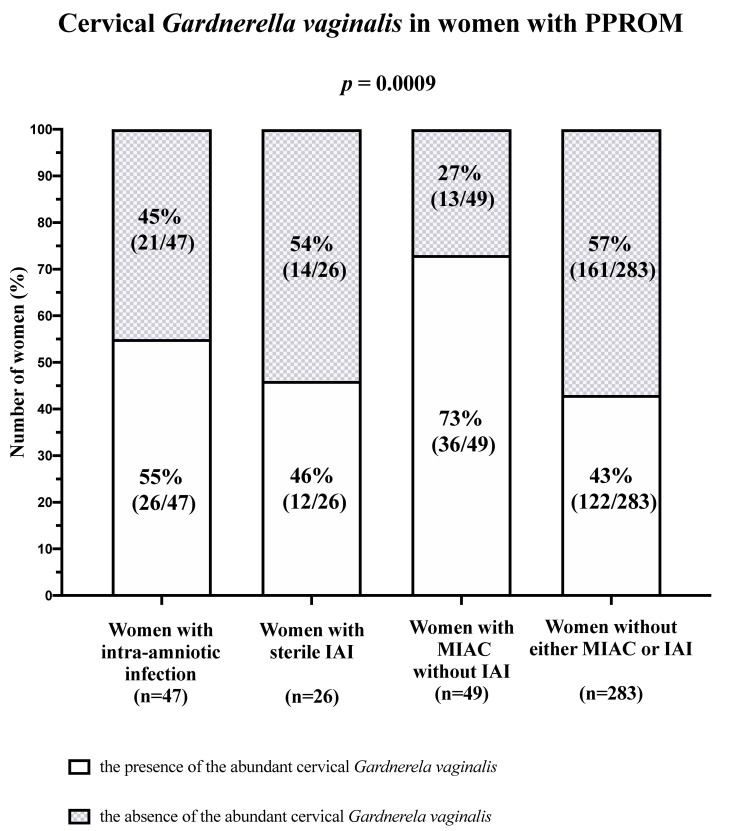
The presence of abundant cervical *G*. *vaginalis* (cervical microbial loads of *G*. *vaginalis* DNA > 1% of amount of the cervical bacterial DNA) in the women with preterm prelabor rupture of the membranes with respect to the presence of microbial invasion of the amniotic cavity and/or intra-amniotic inflammation. A difference in the rates of the abundant cervical *G*. *vaginalis* was identified among the women with intra-amniotic infection, sterile IAI, MIAC without IAI, and without either MIAC or IAI (*p* = 0.0009). Abbreviations: IAI, intra-amniotic inflammation; MIAC, microbial invasion of the amniotic cavity.

A difference in the rates of the abundant cervical *G*. *vaginalis* was identified among the women with intra-amniotic infection, sterile IAI, MIAC without IAI, and without either MIAC or IAI in the crude analysis [intra-amniotic infection 55% (26/47), sterile IAI 46% (12/26), MIAC without IAI 73% (36/49), and without either MIAC or IAI 43% (122/283); *p* = 0.0009; Fig 4], as well as after the adjustment for the different methods of IL-6 assessment (*p* = 0.04).

### The presence of MIAC without IAI and cervical G. vaginalis DNA

Among women with the presence of cervical *G*. *vaginalis* DNA along with a higher cervical load of *G*. *vaginalis* DNA, those with MIAC without IAI had a higher load of the cervical *G*. *vaginalis* DNA than remaining women in the crude analysis (MIAC without IAI: median 2.0 × 10^4^ copies DNA/mL, IQR 1.9 × 10^2^–5.2 × 10^5^ vs. remaining women: median 4.0 × 10^3^ copies DNA/mL, IQR 2.7 × 10^2^–2.1 × 10^5^; *p* = 0.04; Fig 2C) but not after the adjustment for the different methods of IL-6 assessment (*p* = 0.48).

Women with MIAC without IAI had a higher rate of presence of the abundant cervical *G*. *vaginalis* than remaining women in both crude and adjusted analyses [MIAC without IAI 73% (36/49) vs. remaining women 45% (160/356); *p* = 0.0002; adjusted *p* < 0.0001].

### Short-term neonatal morbidity and cervical G. vaginalis

The presence of the abundant cervical *G*. *vaginalis* was not associated with the differences in the short-term neonatal morbidity (Table 3).

**Table 3:** Selected aspects of short-term neonatal morbidity with respect to the presence or absence of the abundant cervical *Gardnerella vaginalis* in women with preterm prelabor rupture of membranes.

	The presence (n = 197)	The absence (n = 208)	*p*-value
Transient tachypnea of newborns	7 (4%)	10 (5%)	0.63
Respiratory distress syndrome	46 (23%)	44 (21%)	0.63
Respiratory disorders	53 (27%)	54 (26%)	0.78
Bronchopulmonary dysplasia	16 (8%)	11 (5%)	0.32
Need for intubation	6 (3%)	7 (3%)	1.00
Intraventricular hemorrhage	24 (12%)	22 (11%)	0.64
Intraventricular hemorrhage (I-II)	23 (12%)	22 (11%)	0.75
Retinopathy of prematurity	5 (3%)	4 (2%)	0.74
Early-onset sepsis	7 (4%)	4 (2%)	0.37
Late-onset sepsis	1 (0.5%)	5 (2%)	0.22
Compound neonatal morbidity	65 (33%)	68 (33%)	0.92
Neonatal death	3 (2%)	2 (1%)	0.68

## Discussion

The principal findings of this study are as follows: i) the cervical *G*. *vaginalis* DNA was found in 94% of women with PPROM between gestational ages 24+0 and 36+6 weeks; ii) the abundant cervical *G*. *vaginalis* DNA was identified in 49% of women with PPROM between gestational ages 24+0 and 36+6 weeks; iii) women with MIAC had a higher microbial load of cervical *G*. *vaginalis* DNA and a higher rate of the abundant cervical *G*. *vaginalis*; iv) the presence of MIAC without IAI was associated with a higher microbial load of cervical *G*. *vaginalis* DNA and a higher rate of the abundant cervical *G*. *vaginalis*; and v) no association between a short-term neonatal morbidity and the abundant cervical *G*. *vaginalis* was revealed.

*G*. *vaginalis* is one of the essential bacteria associated with bacterial vaginosis [17, 35]. Its vaginal presence during pregnancy seems to be common. The prevalence of the vaginal presence of *G*. *vaginalis* DNA differs among the studies, from 27% to 94% of the pregnant women [36–38]. Since an association between bacterial vaginosis and PPROM is well established [19–21, 39], a higher cervical presence of *G*. *vaginalis* among women with PPROM was expected. In this study, RT- PCR was used to detect *G*. *vaginalis* DNA. This method was able identify the cervical *G*. *vaginalis* DNA at very low loads owing to a detection limit of this method between 1 and 10 copies of the nucleic acid. Because of this, the presence of cervical *G*. *vaginalis* DNA was identified in almost in all women (94%) with PPROM. However, 30% (115/379) of the women with proven cervical *G*. *vaginalis* DNA had very low loads (below 1000 copies DNA/mL) of *G*. *vaginalis* DNA. A clinical relevance for such extremely low loads of cervical *G*. *vaginalis* is highly unlikely, and the abundant presence (> 1% of all cervical bacterial DNA) of cervical *G*. *vaginalis* was also taken into consideration in this study. Given this more clinically relevant and meaningful criterion for the cervical presence of *G*. *vaginalis*, the finding regarding the abundant presence of cervical *G*. *vaginalis* (49%) was in line with our previous study [15], where *G*. *vaginalis* DNA identified using 16S rRNA gene sequencing by pyrosequencing, was found in 46% (28/61) of the women with PPROM [15].

MIAC in pregnancies in PPROM represents a very heterogeneous condition due to different: i) microorganisms present in the amniotic fluid [25, 30, 40]; ii) number of microbial species identified in the amniotic fluid [14, 30, 40]; iii) loads of microorganisms revealed in the amniotic fluid [41, 42]; and iv) an intensity of intra-amniotic inflammatory response [25, 31, 40, 41]. Furthermore, MIAC in PPROM has two different clinical phenotypes: i) intra-amniotic infection and ii) the presence of MIAC without IAI [7]. The latter has been shown to have similar intensities of intra-amniotic [7], maternal [8, 9], and fetal [43] inflammatory responses to those without MIAC or IAI. Regardless, the fact that a spectrum of the microorganisms revealed in amniotic fluid from women with PPROM with intra-amniotic infection and with MIAC without IAI looks very similar, their microbial loads seem to be lower in the presence of MIAC without IAI, at least in those with *Ureaplasma* spp. in the amniotic fluid [7]. Moreover, intra-amniotic infection is associated with the most intense intra-amniotic [7], maternal [8, 9], and fetal [43] inflammatory responses.

In this study, we found that the presence of MIAC, but not IAI, was associated with higher abundant cervical *G*. *vaginalis* DNA presence. Interestingly, when women with PPROM were divided into four subgroups (intra-amniotic infection, sterile IAI, MIAC without IAI, and without either MIAC or IAI), the loads of *G*. *vaginalis* DNA and the rate of the abundant cervical presence of *G*. *vaginalis* were the highest in women with MIAC without IAI. This clinically interesting and relevant finding suggests that the presence of the cervical *G*. *vaginalis* in women with PPROM is associated mainly with the development of the less threatening form of MIAC, which does not provoke an inflammatory response in the maternal, fetal, and intra-amniotic compartments.

*G*. *vaginalis* is a pathogen that exhibits the following important virulence determinants: i) adhesion to vaginal epithelial cells [44]; ii) production of biofilms that can be detected not only in the vagina [17, 44] but also in the endometrium and fallopian tubes [45]; and iii) cytotoxic activity [46, 47]. These findings confirm a unique role of *G*. *vaginalis* in the pathophysiology of bacterial vaginosis [35]. The ability of *G*. *vaginalis* to create a biofilm might explain our observation regarding the association between the cervical presence of *G*. *vaginalis* and the presence of MIAC without IAI. It has been shown that some uropathogens such *Escherichia coli* and *Enterococcus faecalis* can enter into the *G*. *vaginalis* biofilm, survive in this environment, and they can continue to ascend in the upper part of the urogenital system, like the bladder [48, 49]. Therefore, we hypothesize that similar mechanism can enhance an ascension of microorganisms with either low virulence potential or with low loads (not enough to trigger an intensive intra-amniotic inflammatory response), from the vagina through the cervix into the amniotic cavity, in pregnancies with PPROM. At this stage we do not have an exact mechanistic explanation of the association between cervical G. vaginalis DNA and the presence of MIAC without IAI. Nevertheless, this observation requires further investigation using larger cohorts of women with very well defined microbial and inflammatory compositions of the cervical and amniotic fluid compartments.

There is evidence that the cervical/vaginal presence of some bacteria might affect some aspects of short-term neonatal morbidity, mainly early-onset sepsis, in newborns from PPROM pregnancies [14, 50]. For example, Brown et al. have shown that vaginal microbiota enriched for *Catonella* spp. and *Sneathia* spp. increased the risk of early-onset sepsis in the newborn [50]. Recently, our group has revealed a relation between the absence of *L*. *crispatus*-dominated cervical microbiota and early-onset sepsis of the newborn [14]. Therefore, selected aspects of short-term neonatal morbidity in relation to the abundant presence of the cervical *G*. *vaginalis* were investigated in this study. However, no associations between the abundant cervical presence of *G*. *vaginalis* and early-onset sepsis and the other selected aspects of short-term neonatal morbidity were identified. This observation was complementary to our previous finding that *L*. *iners*-dominated cervical microbiota did not affect the short-term neonatal morbidity [14].

The main strength of this study was a large cohort of the women with singleton pregnancy complicated by PPROM, for whom the results from amniotic fluid for MIAC and IAI at the time of admission were known. However, this study has some limitations. In the murine model, it has been shown that vaginal infection by *G*. *vaginalis* can lead to an ascending intrauterine infection by this bacteria, mainly in the cases with higher vaginal microbial loads of *G*. *vaginalis* [51]. Nevertheless, the ability of *G*. *vaginalis* to ascend into the uterus has not been demonstrated in the pregnant murine model [52]. Despite the fact that amniotic and cervical fluid samples used in this study were obtained at the time of admission into the labor room, we were unable to use these paired samples for a more comprehensive assessment of the association between the cervical and amniotic fluid presence of *G*. *vaginalis*, owing to the different analytical approaches used to identify *G*. *vaginalis* in both types of the samples. Second, leaking amniotic fluid through the cervix is an unavoidable confounder in PPROM pregnancies that may have affected the obtained cervical fluid samples in the following manner: i) inhibition of the growth of some bacteria [53, 54]; ii) dilution of the cervical fluid samples and a decrease in the load of *G*. *vaginalis* DNA; and iii) elevation of the cervical bacterial DNA loads in the samples from the women with proven MIAC. Third, *G*. *vaginalis* is a bacteria with known phenotypic and genetic heterogeneity [55]. Based on the 473 *G*. *vaginalis* genes, and by sequencing a single 552 bp region of the chaperonin-60 gene, four clades (1–4) and four subgroups (A-D) of *G*. *vaginalis* having different pathogenic potentials were revealed [56–61]. The design of this study did not give us an opportunity to take heterogeneity of *G*. *vaginalis* into consideration. Fourth, data on Nugent score or the presence of Amsel’s clinical criteria for bacterial vaginosis is not available for this cohort of the women. Fifth, the absence of the control cohort consisting from women with an uncomplicated singleton pregnancy, matched for gestational age at sampling, prevented us from the evaluation of the association between the presence of cervical *G*. *vaginalis* DNA and PPROM. In addition, the population of the women with PPROM used in this study was very homogenous involving just Caucasian women without comorbidities. This fact should be considered as a shortcoming due to limited generalization of these results on populations with broad racial and ethnic diversities. Next, two different methods were used in this study to assess amniotic fluid levels of IL-6 (with different cut-off values for IAI) [22, 25]. This fact prevented us from evaluation of the relationship between loads of the cervical *G*. *vaginalis* DNA or an abundance of the cervical *G*. *vaginalis* and the intensity of intra-amniotic inflammation. In addition, the use of two different methods to assess levels of amniotic fluid IL-6 should be taken as a possible confounder. Therefore, appropriate results were adjusted for the different methods of IL-6 assessment. After the adjustment, differences in the rates of the abundant cervical *G*. *vaginalis* among the women with intra-amniotic infection, sterile IAI, MIAC without IAI and without either MIAC or IAI and between the women with MIAC without IAI and remaining women remained statistically significant. However, no difference in the cervical load of *G*. *vaginalis* DNA between women with MIAC without IAI and remaining women was observed after the adjustment for the different methods of IL-6 assessment. Lastly, the presence of MIAC and IAI might be associated with the alteration of the microbial loads of the others cervical microorganisms. Therefore, we cannot rule out that our observations were not a transient effect.

In conclusion, in women with PPROM, the presence of cervical *G*. *vaginalis* was associated with MIAC, mainly without the concurrent presence of IAI.

## Supporting information

S1 Data (XLSX)
